# Conocimiento, creencias y aceptabilidad de la vacuna contra el Virus del Papiloma Humano en padres de Chihuahua, México*

**DOI:** 10.15649/cuidarte.3160

**Published:** 2023-12-24

**Authors:** Rosa Daniela Sánchez Mendoza, Claudia Orozco Gómez, Marily Daniela Amaro Hinojosa, Vicente Jiménez Vázquez

**Affiliations:** 1 . Universidad Autónoma de Chihuahua. Chihuahua, México. Email: dany63355@gmail.com Universidad Autónoma de Chihuahua Universidad Autónoma de Chihuahua Chihuahua Mexico dany63355@gmail.com; 2 . Universidad Autónoma de Chihuahua. Chihuahua, México. Email: clorozco@uach.mx Universidad Autónoma de Chihuahua Universidad Autónoma de Chihuahua Chihuahua Mexico clorozco@uach.mx; 3 . Universidad Autónoma de Chihuahua. Chihuahua, México. Email: damaro@uach.mx Universidad Autónoma de Chihuahua Universidad Autónoma de Chihuahua Chihuahua Mexico damaro@uach.mx; 4 . Universidad Autónoma de Chihuahua. Chihuahua, México. Email: vvazquez@uach.mx Universidad Autónoma de Chihuahua Universidad Autónoma de Chihuahua Chihuahua Mexico vvazquez@uach.mx

**Keywords:** Vacunas contra Papilomavirus, Padres, Conocimiento, Aceptación de la Atención de Salud, Papillomavirus Vaccines, Parents, Knowledge, Patient Acceptance of Health Care, Vacinas contra Papillomavirus, Pais, Conhecimento, Aceitagáo pelo Paciente de Cuidados de Saúde

## Abstract

**Introducción:**

La cobertura de vacunación contra el virus del papiloma humano no se ha realizado en la totalidad de la población, existen factores que interfieren en que los padres de las adolescentes acepten su aplicación.

**Objetivo:**

Relacionar el conocimiento sobre el virus del papiloma humano, el conocimiento sobre la vacuna contra el virus, las creencias sobre la vacuna con la aceptabilidad de la vacuna por los padres de las adolescentes de 9-12 años escolarizadas en Chihuahua, México.

**Materiales y Métodos:**

Estudio de tipo descriptivo, correlacional y transversal, la muestra fue de tipo censal, se conformó por 145 padres de niñas entre 9 a 12 años inscritas en tres primarias públicas ubicadas en una zona urbana de Chihuahua, México.

**Resultados:**

El conocimiento sobre el virus del papiloma humano se relacionó con la aceptabilidad de la vacuna (p < 0,009), de igual manera con el conocimiento acerca de la vacuna del virus del papiloma humano (p < 0,030) mientras que las creencias sobre el VPH y la vacuna no se relacionaron (p < 0,747).

**Discusión:**

Los resultados coinciden con literatura previa en que el conocimiento sobre el virus y su vacuna es bajo, sin embargo, en este estudio las puntuaciones fueron más bajas. Mientras que la aceptabilidad de la vacuna contra el VPH tiende a ser alta al igual que estudios previos.

**Conclusiones:**

El conocimiento sobre el virus del papiloma humano y la vacuna se relacionaron con la aceptabilidad de los padres para aplicar la vacuna a sus hijas.

## Abstract (en)

**Introduction:**

Human papillomavirus vaccination coverage has not been achieved in the general population. There are factors that interfere with the acceptance of the vaccine by the parents of adolescent girls.

**Objective:**

To correlate knowledge of human papillomavirus, knowledge of the vaccine against the virus, and beliefs about the vaccine with vaccine acceptance among parents of adolescent girls aged 9-12 years in Chihuahua, Mexico.

**Materials and Methods:**

A descriptive, correlational, and cross-sectional study was conducted with a census sample of 145 parents of girls between the ages of 9 and 12 enrolled in three public elementary schools in an urban area of Chihuahua, Mexico.

**Results:**

Knowledge of human papillomavirus was related to vaccine acceptance (p < 0.009), as was knowledge of the human papillomavirus vaccine (p < 0.030). In contrast, beliefs about HPV and the vaccine were not related (p < 0.747).

**Discussion:**

The results are consistent with previous literature in that knowledge of the virus and its vaccine is low, but the scores were lower in this study. In contrast, HPV vaccine acceptance tends to be high, as in previous studies.

**Conclusions:**

Knowledge about human papillomavirus and the vaccine was associated with parental acceptance of giving it to their daughters.

## Resumo

**Introdujo:**

A cobertura vacinal contra o papilomavírus humano nao tem sido realizada em toda a populagáo, existem fatores que interferem na aceitagáo da sua aplicagáo pelos pais de meninas adolescentes.

**Objetivo:**

Relacionar o conhecimento sobre o papilomavírus humano, o conhecimento sobre a vacina contra o virus, as crengas sobre a vacina com a aceitabilidade da vacina pelos pais de meninas adolescentes de 9 a 12 anos que frequentam a escola em Chihuahua, México.

**Materiais e Métodos:**

Estudo descritivo, correlacional e transversal, a amostra foi do tipo censitária, composta por 145 pais de meninas entre 9 e 12 anos matriculadas em tres escolas primárias públicas localizadas em uma área urbana de Chihuahua, México.

**Resultados:**

O conhecimento sobre o papilomavírus humano esteve relacionado com a aceitabilidade da vacina (p < 0,009), da mesma forma com o conhecimento sobre a vacina contra o papilomavírus humano (p < 0,030), enquanto as crengas sobre o HPV e a vacina náo foram relacionadas (p < 0,747).

**Discussao:**

Os resultados coincidem com a literatura anterior na medida em que o conhecimento sobre o vírus e sua vacina é baixo, porém, neste estudo as pontuagóes foram inferiores. Embora a aceitabilidade da vacina contra o HPV tenda a ser elevada como em estudos anteriores.

**Condusdes:**

O conhecimento sobre o papilomavírus humano e a vacina esteve relacionado a aceitabilidade dos pais em aplicar a vacina em suas filhas.

## Introducción

El Virus del Papiloma Humano (VPH) es una enfermedad de transmisión sexual muy frecuente y constituye un problema de salud pública^1^^,^^2^. Existen dos grupos de VPH de transmisión sexual: de bajo y alto riesgo, los primeros son responsables de producir verrugas en el área de los genitales, el ano, la boca o la garganta^3^, mientras que el VPH de alto riesgo causan cerca del 5% de todos los cánceres en el mundo, y son responsables del 90% de los cánceres de ano y cuello uterino, cerca de 70% de los cánceres de vagina, de vulva y orofaríngeo y más del 60% de los cánceres de pene^4^.

A nivel mundial, en el 2020 se estimaron 432 000 muertes a causa del cáncer cervicouterino, con una incidencia de 604 000 casos nuevos, de los cuales alrededor del 90% se produjeron en países de bajos y medianos ingresos^5^, mientras que en América Latina es la segunda neoplasia más común, y en México es la segunda causa de muerte por cáncer en la mujer, anualmente se estima una ocurrencia de 13 960 casos en mujeres, con una incidencia de 23,3 casos por 100 000 mujeres^6^.

La detección temprana del VPH es uno de los métodos que favorecen la prevención del cáncer cervicouterino^7^ y la vacunación contra el VPH es la forma más efectiva a largo plazo.^8^ La población blanco para la administración de la vacuna contra el VPH en esquema de una o dos dosis son las niñas entre 9 a 14 años previo al debut sexual. Sin embargo, esta puede aplicarse en niñas mayores de 15 años, así como los niños, siempre y cuando sea asequible y no desvíe recursos de la vacunación de la población objetivo principal^9^. La Administración de Alimentos y Medicamentos ha aprobado tres vacunas para la prevención de la infección por VPH: Gardasil (tetravalente), Gardasil 9 (nonavalente) y Cervarix (bivalente), cabe señalar que las tres vacunas protegen contra la infección de VPH tipo 16 y 18, los que se consideran los principales causantes de desarrollo de cáncer cervicouterino^10^.

La vacuna contra el VPH se aplica en 67% de los estados miembros de la Organización Mundial de la Salud y se calcula que, a finales del 2021 la cobertura mundial de la dosis final fue de 16%^11^. Cabe señalar que su cobertura ha disminuido a nivel global, del año 2019 al 2021, la cobertura de la primera dosis de la vacuna contra el VPH se redujo entre un 25 % y un 15 %, lo que equivale a 3,5 millones de niñas que no la recibieron^9^. En las regiones más desarrolladas del mundo 33,6% de las mujeres de 10 a 20 años completó el esquema de vacunación, mientras que, en las regiones menos desarrolladas, en las que se incluye México la cobertura fue de 2,7 %^12^. La cobertura global media de vacunación contra el VPH en América Latina para al menos una dosis es del 80%, mientras que el 55% corresponde para el programa de dos dosis completo^13^.

La literatura refiere que la falta de cobertura se debe a la escasez de producción de la vacuna, principalmente durante la pandemia por Covid-19, fallas en los servicios de atención primaria, disminución de la demanda^14^ y la aceptabilidad. En este sentido se ha documentado que la aceptabilidad de la vacuna por parte de los padres es baja^15^^,^^16^, entendiéndose por aceptación de la vacuna contra el VPH, la intención voluntaria de recibir una vacuna o expresión de acuerdo de que la vacuna representa una buena estrategia preventiva^17^. Los padres son los principales implicados en la aceptabilidad de los cuidados que el profesional de salud otorga a sus hijos, así como la realización de cualquier procedimiento, incluido la aplicación de la vacuna del VPH^18^. Además, existen factores que se relacionan para que se adquiera o acepte la vacuna contra el VPH por parte de los padres o madres de las adolescentes, las cuales están cifradas de mala información adquirida por sus creencias, especulaciones falsas o tabús^19^, así como el escaso conocimiento sobre la vacuna contra el VPH, en donde se evidencia que a menor conocimiento menor es la aceptabilidad por parte de los padres^20^, ^21^, ^22^,^15^, ^23^^).^_._

Otro factor que influye en la aceptabilidad son la creencias relacionadas con la vacuna, entre estas destaca que el aceptar la vacuna contra el VPH para sus hijas va a inducirlas a un inicio temprano de relaciones sexuales y además, de generarles una supuesta seguridad que las llevará a tener múltiples compañeros sexuales, libertinaje sexual y menos precauciones durante el acto sexual^24^, también los padres desconfían de la seguridad de la vacuna, ya que afirman que la industria farmacéutica no es transparente y presentan desconfianza^25^ y tienen miedo a los efectos adversos que sus hijos pudieran tener al recibir la vacuna contra el VPH^24^^-^^25^.

La evidencia existente sobre la aceptabilidad, el conocimiento sobre el VPH, conocimiento sobre la vacuna contra el VPH y las creencias surgió de estudios realizados en Perú, Colombia, Argentina y México^20^^,^^21^^,^^15^^,^^16^^,^^22^^,^^23^. Sin embargo, poco se han estudiado estas variables en conjunto, es por ello que el objetivo de este estudio fue analizar la relación entre el conocimiento sobre el VPH, el conocimiento sobre la vacuna contra el VPH, las creencias sobre el VPH y la vacuna con la aceptabilidad de la vacuna contra el VPH por los padres de las adolescentes de 9-12 años escolarizadas en Chihuahua, México.

## Materiales y Métodos

Este estudio fue descriptivo, correlacional y transversal. La muestra fue de tipo censal la cual se conformó por 145 padres de familia (143 madres y 2 padres) de adolescentes mujeres de 9 a 12 años, inscritas en tres primarias públicas, ubicadas en una zona urbana de Chihuahua, México, entre los meses de junio a noviembre del 2022. Se incluyeron a madres y padres de familia que desearon participar en el estudio; se eliminaron aquellos instrumentos que se encontraban incompletos en los ítems de los cuestionarios.

Para obtener la información se utilizó una cédula de datos personales, preguntas para conocer aspectos sobre los antecedentes de prevención de cáncer cervicouterino de la madre, también se incluyeron preguntas para conocer las fuentes de donde ha obtenido información sobre el VPH. Para medir el conocimiento del VPH y la vacuna contra el VPH se emplearon 23 preguntas de la escala de conocimiento general de VPH^26^. Para cada respuesta correcta se otorgó valor de 1 y 0 por cada respuesta incorrecta o "No sé". Como tal, la puntuación general del conocimiento del VPH podría oscilar entre 0 a 16 y del conocimiento de la vacuna del VPH de 0 a 7. A mayor puntuación se considerará mayor conocimiento. El instrumento en este estudio mostró confiabilidad aceptable *(a=* 0,77).

Para medir la aceptabilidad y las creencias sobre el VPH y la vacuna se utilizaron las subescalas de creencias (16 preguntas) y aceptabilidad de la vacuna del VPH (9 preguntas) del cuestionario de conocimiento, creencias y aceptación de la vacuna^27^, las cuales han sido validadas en población Mexicana^28^. Estas tienen opción de respuesta tipo Likert que va de 1 = “Muy en desacuerdo” a 4= “Muy de acuerdo”. A mayor puntuación mayor aceptabilidad de las madres y padres para que sus hijas reciban la vacuna contra el VPH y a mayor puntuación mayores creencias erróneas sobre el VPH y la vacuna. La confiabilidad encontrada de estas subescalas en este estudio fueron a= 0,64 y a= 0,77, respectivamente.

Para la recolección de la información se realizó una primera visita a las escuelas primarias previstas para conocer el número exacto de la población y pedir permiso a las autoridades directivas para la aplicación de los instrumentos y acordar la fecha en que se podría acudir. Se abordó a la madre o padre de familia al finalizar las reuniones informativas de rutina, en los días y horarios establecidos tanto por las autoridades educativas y el personal docente. Inicialmente se les invitó a participar, para quienes desearon participar se realizó la lectura del consentimiento informado de forma grupal y procedieron a firmarlo. Luego, se les hizo entrega de los cuestionarios, los cuales leían de forma individual, durante el llenado se aclararon dudas para contestar los cuestionarios, finalmente se agradeció su participación.

La información recabada fue procesada a través del paquete estadístico Statistical Package for Social Sciences versión 25 para Microsoft y se almacenó en Figshare^29^. Se calcularon frecuencias y porcentajes, se hizo uso de la estadística descriptiva utilizando media, mediana y desviación estándar. También se determinó la distribución de la normalidad a través de la prueba de Kolmogorov Smirnov, en donde no se encontró normalidad de los datos por lo que se hizo uso del coeficiente de correlación de Spearman.

Para fines éticos se apegó a lo descrito en el Reglamento de la Ley General de Salud en Materia de Investigación para la Salud^30^, se dio libertad de cancelar la participación del participante en el momento que lo deseará, se mantuvo la confidencialidad y anonimato de la información. También se contó con la aprobación del Comité de Ética en Investigación en investigación de la Facultad de Enfermería y Nutriología de la Universidad Autónoma de Chihuahua con el folio: SIP-CI-M/2022/01.

## Resultados

La edad promedio de los participantes fue 35,65 años (DE = 6,44 Min 21 Max 57). En la Tabla 1 se observa que en su mayoría los participantes fueron madres, con un nivel de estudio de secundaria, la ocupación predominante fue empleadas, se encuentran casadas y en su mayoría profesan la religión católica.

**Tabla 1:** Características sociodemográficas de los participantes

Características	N (145)	%
Sexo		
Femenino	143	98,60
Masculino	2	1,30
Nivel de Estudio		
Primaria	18	12,40
Secundaria	64	44,10
Preparatoria	38	26,20
Universidad	18	12,40
Otro	7	4,80
Ocupación		
Desempleada (o)	37	25,50
Servidor público	3	2,10
Comerciante	11	7,60
Empleada (o)	58	40,00
Otro	36	24,80
Estado Civil		
Soltera (o)	21	14,50
Casada (o)	69	47,60
Divorciada (o)	16	11,00
Viuda (o)	6	4,10
Unión Libre	33	22,80
Religión		
Católica	109	75,20
Testigo de Jehová	1	0,70
Evangélica	10	6,90
Otro	5	3,40
Ninguna	20	13,80
Ingreso Familiar Mensual		
Menos de $3,000	39	26,90
$3,000-$10,999	79	54,50
$11,000-$16,999	13	9,00
$17,000-$23,999	3	2,10
Más de $24,000	3	2,10
Sin dato	8	5,50

*Nota: n = tamaño de la muestra; % = porcentaje; n = 145*

Las madres y padres refirieron que ninguna de sus hijas había recibido la vacuna contra el VPH, el 73,10% (106) de sus hijas contaban con la aplicación del esquema completo de otras vacunas correspondientes a su edad, 16,60% (24) contaba con solo algunas vacunas y 10,40%(15), no estaba segura de que tuviera completo el esquema. En la Tabla 2 se observa que 16,10% de las participantes del sexo femenino contaban con antecedentes de cáncer cervicouterino, 86% mencionaron haberse realizado el Papanicolaou. Respecto a si se habían aplicado la vacuna contra el VPH 19,60% recordaban haberse aplicado la vacuna del VPH, en su mayoría habían recibido alguna dosis de la vacuna contra el VPH.

**Tabla 2:** Antecedentes patológicos y de conductas de prevención del VPH maternos

Características	n	%
Antecedentes de CCU		
No	120	83,90
Si	23	16,10
¿Quién ha tenido CUU?*		
Madre de familia	6	26,10
Hermana	1	4,30
Abuela	9	39,10
Tía	7	30,40
Características	n	%
Realizado prueba de Papanicolaou		
Nunca	20	14,00
Sí	123	86,00
Aplicación de la vacuna contra el VPH		
Sí	28	19,60
No	93	65,00
No lo sé	22	15,40
Número de dosis**		
Una dosis	9	32,10
Dos dosis	10	35,70
Tres dosis	1	3,60
No sé	8	28,60

Nota: CUU = Cáncer cervicouterino; n = tamaño de muestra; % = porcentaje; n* = 23; n**=28

Se encontró que más de la mitad de los participantes alguna vez han recibido información del VPH y de la vacuna contra el VPH, los medios de comunicación fue la principal fuente de información, 47,60% mencionaron que un médico o profesional de enfermería le ha informado de la vacuna contra el VPH (Tabla 3).

**Tabla 3:** Fuentes de información del VPH en los padres

Pregunta	Si		No	
	n	%	n	%
¿Alguna vez ha oído hablar del VPH?	126	86,90	19	13,10
¿Alguna vez ha recibido información acerca del VPH?	100	69,00	45	31,00
¿Alguna vez un médico/enfermero le ha informado acerca del VPH?	76	52,40	69	47,60
Otras fuentes de información sobre el VPH	n	%		
Amigos	10	6,90		
Familiares	10	6,90		
Personal escolar	16	11,00		
Medios de comunicación	90	62,10		
No he escuchado	19	13,10		
		Si		No
	n	%	n	%
¿Alguna vez ha oído hablar de la vacuna contra el VPH?	117	80,70	28	19,30
¿Alguna vez ha recibido información de la vacuna contra el VPH?	83	57,20	62	42,80
¿Alguna vez un médico/enfermero le ha informado de la vacuna contra el VPH?	69	47,60	76	52,40
Amigos	9	6,20		
Familiares	16	11,00		
Personal escolar	17	11,70		
Medios de comunicación	75	51,70		
No he escuchado	28	19,40		

*n = tamaño de la muestra; % = porcentaje; n = 145*

Respecto a las variables de estudio, en la Tabla 4 se observa que el conocimiento general presentó un promedio de 22,12 (DE= 16, 53), el conocimiento sobre la vacuna contra el VPH fue mayor que el conocimiento sobre el VPH (X= 27, 29 Vs *IX =* 19,87). Las creencias sobre el VPH y la vacuna mostraron un promedio de 53,53 (DE = 9,35). Por otra parte, la puntuación de la aceptabilidad de los padres de familia de la vacuna contra el VPH se situó ligeramente por arriba de la media (X= 68,32, DE = 15,10).

**Tabla 4:** Estadística descriptiva de variables de estudio

Índice	*X*	Mdn	DE	Mín	Máx
Conocimiento general	22,12	21,73	16,53	0	56,52
Conocimiento del VPH	19,87	18,75	15,75	0	50,00
Conocimiento de la vacuna contra el VPH	27,29	28,57	23,32	0	100
Creencias sobre el VPH y la vacuna	53,53	54,16	9,35	22,92	100
Aceptabilidad de la vacuna del VPH	68,32	66,66	15,10	0	100

*Nota:X̅ = Media aritmetíca; Mdn = Mediana, DE = Desviación estándar; Mín =valor minímo; Máx = Valor máximo; n = 145*

Al efectuar la correlación de Spearman se encontró una relación positiva y significativa entre conocimiento del VPH y el conocimiento de la vacuna del VPH con la aceptabilidad de la vacuna. Las creencias sobre el VPH y la vacuna no se relacionaron significativamente con la aceptabilidad de la vacuna (Tabla 5).

**Tabla 5:** Relación del conocimiento del VPH, conocimiento de la vacuna del VPH y creencias sobre el VPH y la vacuna con la aceptabilidad de la vacuna

Variables	Aceptabilidad	
	r	^p^
Conocimiento del VPH	0,215**	0,009
Conocimiento de la vacuna del VPH	0,180*	0,030
Creencias sobre el VPH y la vacuna	0,027	0,747

Nota: ** p<.01, *p<.05, n=145, r=coeficiente de correlación de Spearman

## Discusión

En este estudio dentro de los hallazgos sociodemográficos se encontró que predomina la población femenina en comparación con la población masculina, lo que evidencia que las mujeres son quienes ejercen mayor responsabilidad en el cuidado de sus hijos, semejante a lo encontrado en otros estudios^31^, ^23^, sin embargo, es importante que la figura paterna se involucre en el cuidado de sus hijos, debido a que se ha documentado que esto beneficia al desarrollo y bienestar de estos^32^.

Las hijas de los participantes del estudio no se habían vacunado contra el VPH, sin embargo, la mayoría contaban con aplicación previa de otras vacunas, lo cual hace pensar que esto podría influir en la aceptabilidad de la vacuna contra el VPH. Además, el que las madres hayan recibido al menos alguna dosis de la vacuna contra el VPH podría aumentar la probabilidad de la aceptación de la vacuna contra el VPH en sus hijas. Cabe señalar que en las madres del estudio se encontró falta de adherencia al esquema completo de vacunación contra el VPH, estos hallazgos son similares a estudios previos^33^, ^34^, por lo que es importante que, se le dé seguimiento para tener el esquema completo y de esta manera adquieran una mejor protección.

Gran parte de los participantes habían escuchado hablar del VPH y su vacuna en comparación con el estudio realizado por He y He^35^ en China y Jurado y Acosta^21^ en Argentina donde mencionan que menos de la mitad de las madres o padres habían escuchado hablar del VPH. Pese a que el profesional de salud juega un papel importante en proporcionar información a la población, en este estudio no son la principal fuente, diferente a lo expresado por Medina- Fernández y cols^36^ y Luna-Chairez y cols^15^, en Querétaro y Chihuahua respectivamente, donde se menciona que las fuentes de obtención de información provienen de la clínica de salud y el personal de salud principalmente. De igual forma un estudio realizado por Jean-Frangois^37^ en Francia, evidencia que los médicos tratantes y el médico escolar fueron las dos fuentes de información más citadas, esto pudiera ser por la presencia de personal de salud en el ámbito escolar, lo cual en la mayor parte del contexto mexicano no se cuenta con un profesional de enfermería escolar, sino que acude de manera esporádica a realizar acciones de promoción de la salud.

En este estudio un alto porcentaje de los padres y madres adquieren información a través de los medios de comunicación, sin embargo, en ocasiones la información sobre las vacunas contra el VPH y el cáncer tanto en medios de comunicación, internet y las redes sociales es incorrecta. Se destaca la necesidad de que los profesionales de la salud sean quienes conversen sobre las preocupaciones de los padres relacionados con la seguridad de la vacuna contra el VPH^38^.

Estudios realizados en Perú^20^ y México^15^ evidenciaron que el conocimiento en los padres es bajo, sin embargo, en este estudio los puntajes del conocimiento tanto del VPH como de la vacuna se observaron más bajos, esto puede deberse a que existe poca difusión sobre el VPH y su vacunación por parte del personal de salud. Asimismo, la escolaridad de la mayoría de los padres es básica, en este sentido existe evidencia que las madres que tienen escolaridad media superior tienen mayor probabilidad de tener menos conocimientos acerca de la vacuna contra el VPH^39^.

Cabe señalar que la información es un factor que estimula las habilidades conductuales que dan como resultado un cambio conductual de prevención de riesgos y sostenimiento del cambio^40^ por lo que proveer de información al padre o madre de los y las adolescentes es esencial para lograr una cobertura suficiente de inmunización, para lo cual se deben realizar estrategias para garantizar que las madres que enfrentan la toma de decisión de vacunar a su hija o hijo puedan hacerlo con una base bien informada y justa^41^.

La aceptabilidad de la vacuna contra el VPH por parte de los padres de familia tiende a ser alta, esto podría relacionarse a que la colecta de información fue en las aulas escolares, mismo lugar donde se realizan las campañas de vacunación de forma gratuita, ya que en México la administración de esta vacuna forma parte del Programa Nacional de Vacunación, por lo que el costo de la vacuna en el grupo blanco de vacunación no se debería considerar como una barrera para su aceptabilidad. En estudios previos realizados en Nigeria, Colombia y Argentina^42^, ^23^, ^21^ se ha observado que se presenta alta aceptabilidad de la vacuna contra el VPH. Cabe mencionar que un estudio realizado en Estados Unidos, se encontró mayor aceptabilidad de los padres para vacunar a sus hijos adolescentes varones^43^, es importante mencionar que en México la población blanco de esta vacuna son las mujeres, lo cual hace pensar que la aceptabilidad de la vacuna contra el VPH pudiera estar condicionada por el género.

Respecto al objetivo del estudio se encontró una relación entre conocimiento del VPH, conocimiento de la vacuna del VPH y la aceptabilidad, caso similar a lo encontrado en diferentes estudios^22^, ^20^, ^21^, ^16^, si bien el conocimiento es un factor que facilita la adopción de una conducta^44^, no tiene por qué ser suficiente para producirlo^45^. Se ha evidenciado que existen otros factores que influyen en que se tenga la intención de realizar una conducta en este caso recibir la vacuna contra el VPH, como la actitud, los beneficio percibidos y las creencias^22^^,^^16^.

Las creencias sobre el VPH y la vacuna tienden a ser erróneas, estas no se relacionaron con la aceptabilidad de la vacuna, caso diferente en lo encontrado en diversos estudios realizados por Chaupis- Zeballos y cols^20^ así por Juntasopeepun y Thana^46^ en Perú y Tailandia en este último las creencias que se relacionaban con la aceptabilidad eran creencias con aspectos religiosos y en este estudio las creencias estaban basadas en la infección, las complicaciones y la seguridad de la vacuna. Es importante continuar informando a la población sobre el VPH y la seguridad de su vacuna debido a que esto se ha identificado como un factor que interviene de manera importante en la aceptabilidad de la vacuna por los efectos adversos que se pudieran generar^24^.

Dentro de las limitaciones del estudio se puede señalar el tamaño de la muestra, por lo que se recomienda efectuar un estudio con mayor número de participantes, para tener mayor representatividad de la población de estudio. Además, pudiera ser que las creencias no fueron medidas con total precisión debido a que la subescala tiene un alpha de Cronbach bajo, cabe señalar que de la versión original es la primera vez que valida en población mexicana, por lo que se recomienda realizar análisis de las preguntas que contribuyen a tener un coeficiente de confidencialidad aceptable o utilizar otras escalas que reporten mayor confiabilidad.

## Conclusiones

El conocimiento tanto del VPH como de la vacuna contra este virus es bajo, pese a esto se relaciona con la aceptabilidad de la vacuna, las creencias sobre el VPH y la vacuna tienden a ser erróneas y estas no se relacionan con la aceptabilidad de la vacuna contra el VPH. Se sugiere crear estrategias por parte de los profesionales de enfermería que permitan fortalecer el programa de vacunación para concientizar a la población sobre la importancia de la vacunación contra el VPH y uso de recursos sanitarios disponibles.

Además de implementar medios de comunicación o redes sociales para favorecer la aceptabilidad de la vacuna en los padres de familia y realizar difusión de las demás medidas de prevención del VPH y sus complicaciones para que esta información sea transmitida a las y los adolescentes. También se sugiere estudiar otros factores o variables sociodemográficas de la madre o padre que pudieran influir en la aceptabilidad de la vacuna, explorar las variables de estudio en ámbitos escolares privados y estudiar la aceptabilidad de los padres de la vacuna contra el VPH en sus hijos hombres para analizar la diferencia por género.
